# Elagolix: a promising oral GnRH antagonist for endometriosis-associated pain

**DOI:** 10.18632/oncotarget.22381

**Published:** 2017-11-11

**Authors:** Erica C. Dun, Hugh S. Taylor

**Affiliations:** Hugh S. Taylor: Department of Obstetrics, Gynecology, and Reproductive Sciences, Yale School of Medicine, New Haven, Connecticut, USA

**Keywords:** elagolix, GnRH antagonist, endometriosis-related pain, endometriosis

Endometriosis is an inflammatory condition that affects 6-10% of reproductive-aged women, and up to 50% of those with chronic pelvic pain. Endometriosis-associated pain is caused by implantation and activation of ectopic endometrium in the peritoneum of pelvic and abdominal viscera [1]. Although surgery has been the route to diagnose and treat endometriosis, medication is increasingly important to help manage its chronic symptoms. Medical management of endometriosis-associated pain has been focused on menstrual cycle suppression with combined oral contraceptives and suppression of inflammatory mediators with nonsteroidal anti-inflammatory drugs (NSAIDs). However, these medications fail in approximately 50% of patients. The next class of medication used for the treatment of endometriosis has traditionally been a gonadotropin releasing hormone (GnRH) agonists that diminish estrogen to postmenopausal levels.

Designing an effective, orally administered, nonpeptide GnRH antagonists has been a prime drug development goal for the treatment of endometriosis-associated pain. The difficulty has been finding a potent drug that, at the same time, limits the severe hypoestrogenic effects and is overall well tolerated. A first generation nonpeptide GnRH antagonist, NBI-42902 was developed and described in 2005, but subsequent human studies showed inhibition of the liver P450 enzymes [2]. Elagolix, a second generation GnRH antagonist, was subsequently developed. Initial evaluation of the drug showed rapid onset of action, good tolerability, and no changes in liver enzymes. In addition, its therapeutic and hypoestrogenic effects are rapidly reversed after cessation of the drug [3, 4]. Further testing in two multicenter, randomized placebo-controlled phase 2 trials showed clinical efficacy and dose-dependent improvement of endometriosis-related dysmenorrhea, dyspareunia, and nonmenstrual pelvic pain. Adverse side effects were similar in nature to other GnRH agonists and antagonists, including nausea, headaches, and hot flushes. However, these the reported adverse side effects were rated only mild to moderate, and <1% of the participants dropped out due to adverse effects. Bone mineral density (BMD) was significantly lower in the elagolix groups versus placebo [5, 6]. However, in a trial of elagolix versus depot medroxyprogesterone acetate, both treatments induced minimal mean changes from baseline in BMD after 24 weeks of treatment and the effects showed a degree of reversal at 24 weeks posttreatment [7].

In a recent publication in the *New England Journal of Medicine,* Taylor *et al.* reported the results of two multicenter double-blind, randomized, placebo-controlled phase 3 trials, Elaris Endometriosis I and II (EM-I and EM-II) [8]. Similar to the phase 2 trials, premenopausal women between the ages of 18 and 49 years with laparoscopically confirmed endometriosis underwent randomization in to 3 groups: elagolix 150mg daily, elagolix 200mg twice a day and placebo. EM-1 was conducted at 151 sites in the United States and Canada from and enrolled 872 women with 652 (74.9%) completing the six-month protocol. EM-2 was conducted at 187 sites in 13 countries and enrolled 817 women with 632 (77.4%) completing the protocol. The protocol included four intervals: a washout of hormonal therapies, screening period of up to 100 days, a 6 month treatment period, and follow-up period of up to 12 months. The primary endpoints were the proportion of subjects who had a clinical response to dysmenorrhea and non-menstrual pelvic pain at 3 months. Secondary endpoints included mean changes in pain score, use of rescue NSAID and opioids, and reporting of safety and adverse effects such as vasomotor symptoms, BMD, lipid changes and endometrial thickness.

The results of EM-I and EM-II showed similar significant, dose-dependent clinical improvement in both dysmenorrhea and nonmenstrual pelvic pain. In terms of dysmenorrhea, the percentage of women with clinical response in EM-I were 46.6%, 75.8% *vs* 19.6% and EM-II were 43.4%, 72.4% *vs* 22.7%, in the lower-dose, higher-dose, and placebo, respectively (*p* < 0.001). In terms of nonmenstrual pelvic pain, EM-I showed clinical response in 50.4%, 54.5% *vs* 36.5% and EM-II 49.8%, 57.8% *vs* 36.5% (*p* = 0.003 and *p* < 0.001). Women in the higher-dose elagolix group use significantly less analgesics (NSAIDs and opioids); however, use remained unchanged in the lower-dose. Discontinuation of the trial due to hot flushes occurred in < 1% of women in the lower-dose group and <3% in the higher-dose. At 6 months, BMD at the lumbar spine, femoral neck, and total hip showed a significant and dose dependent decrease in the elagolix groups versus placebo. The drug produced a dose dependent decrease in endometrial thickness.

The findings of two large multicenter, multinational phase 3 trials consistently showed dose-dependent decreases in dysmenorrhea and nonmenstrual pelvic pain in women with endometriosis-associated pelvic pain. Importantly, elagolix is clinically efficacious at two doses, allowing the drug to be individualized. Other advantages of elagolix include its immediate suppression of pituitary gonadotrophs, avoiding the initial 1-2 week flare-up effect of GnRH agonists and thereby providing immediate therapeutic effect. Its nonpeptide drug structure allows for oral administration, an improvement over intramuscular injections of GnRH peptide agonists and antagonists currently in use; intramuscular injections can cause pain, injection site reactions, and there is no ability to discontinue long acting depot injections if desired. Vasomotor symptoms were described as mild to moderate and are not as severe as seen with currently used GnRH agonist treatment.

Further studies are needed to determine if add-back therapy may lessen the mild to moderate hypoestrogenic side effects and to elucidate the significance of the BMD loss if elagolix is used for long-term treatment in pre-menopausal women. Elagolix is poised to become a revolutionary advancement in the traditionally challenging medical management of endometriosis-associated pain.
